# Diagnostic value of tubular and glomerular biomarkers across different stages of kidney injury in patients with type 2 diabetic nephropathy

**DOI:** 10.3389/fendo.2026.1802069

**Published:** 2026-06-18

**Authors:** Ming-Zhi Xiao, Xin-Xin Pang, Di Niu, Rui-Fang Chen

**Affiliations:** 1 Department of Endocrinology, The Second Clinical Medical College of Henan University of Traditional Chinese Medicine, Zhengzhou, Henan, China; 2 Department of Nephrology, Henan Provincial Hospital of Traditional Chinese Medicine (The Second Affiliated Hospital of Henan University of Traditional Chinese Medicine), Zhengzhou, Henan, China

**Keywords:** diabetic nephropathy, estimated glomerular filtration rate, type 2 diabetes mellitus, urinary biomarkers, urine albumin-to-creatinine ratio

## Abstract

**Background:**

Diabetic nephropathy (DN) remains a major cause of chronic kidney disease in patients with type 2 diabetes mellitus (T2DM), yet conventional markers incompletely capture heterogeneous renal injury. This study aimed to evaluate the diagnostic performance and stage-wise behavior of clinically accessible urinary glomerular and tubular biomarkers for identifying DN.

**Methods:**

This multicenter retrospective observational study enrolled 320 hospitalized patients with T2DM, including 160 patients with DN and 160 age- and sex-matched controls without nephropathy. Kidney function was stratified by estimated glomerular filtration rate (eGFR) categories G1 to G5. Urinary glomerular and tubular biomarkers were compared between groups and across stages. Receiver operating characteristic analysis, multivariable logistic regression, DeLong testing, calibration assessment, bootstrap internal validation, subgroup analysis, and sensitivity analyses were performed.

**Results:**

Both glomerular and tubular biomarkers were significantly elevated in DN, with separation from controls evident even in G1. Urine albumin-to-creatinine ratio (UACR) showed the highest single-marker discrimination for DN (area under the curve 0.93, 95% confidence interval 0.90 to 0.96), followed by urinary albumin (0.91, 0.88 to 0.94) and urinary β2-microglobulin (0.88, 0.84 to 0.92). For stage discrimination, urinary β2-microglobulin performed best (area under the curve 0.85, 95% confidence interval 0.79 to 0.91). A combined model incorporating UACR, urinary β2-microglobulin, and urinary N-acetyl-β-D-glucosaminidase improved discrimination versus UACR alone (area under the curve 0.96 vs 0.93; DeLong Z = 2.74, P = 0.006), with good calibration and stable performance after bootstrap validation and sensitivity analyses.

**Conclusions:**

Clinically available urinary tubular and glomerular biomarkers distinguish DN from matched T2DM controls across Kidney Disease: Improving Global Outcomes (KDIGO) eGFR stages, including preserved eGFR. Integrating tubular injury markers with UACR significantly enhances diagnostic performance beyond UACR alone.

## 1 Introduction

Diabetic nephropathy (DN), a major phenotype within diabetic kidney disease, remains a leading contributor to chronic kidney disease (CKD) and kidney failure and is associated with excess cardiovascular morbidity and mortality. Contemporary CKD frameworks emphasize integrated risk assessment using estimated glomerular filtration rate (eGFR) and albuminuria, both for staging and for guiding monitoring intensity and therapeutic decisions (1). However, clinical practice continues to face several challenges: kidney injury in type 2 diabetes is heterogeneous, eGFR decline may lag behind structural damage, and albuminuria does not fully capture non-albuminuric or mixed patterns of diabetic renal injury. These limitations motivate the continued evaluation of urinary biomarkers that reflect distinct anatomic compartments and pathophysiologic processes, including glomerular permselectivity impairment and proximal tubular dysfunction (2, 3). A growing body of literature supports the premise that tubulointerstitial injury is closely linked to CKD progression and that urine-based markers can provide noninvasive signals of tubular injury, inflammation, and impaired tubular protein handling (4, 5). In DN, urinary biomarkers have been proposed to complement albuminuria by improving early detection, capturing injury pathways not reflected by UACR alone, and refining stage-wise characterization of disease severity (6). Multiplex and panel-based approaches have also been explored to improve diagnostic yield compared with single-marker strategies, reflecting the multifactorial nature of diabetic renal injury (7).

Accurate staging and cross-stage comparisons also depend on the choice and interpretation of GFR estimating equations. The Kidney Disease: Improving Global Outcomes (KDIGO) 2024 CKD guideline and related commentaries underscore the need for validated, standardized eGFR estimation and careful consideration of equation-related reclassification in clinical populations (8). Importantly, not all diabetic kidney disease follows the classic albuminuric trajectory. A subset of patients with diabetes may experience declining kidney function despite normoalbuminuria or the absence of overt albuminuria, a phenotype often referred to as non-albuminuric diabetic kidney disease (9). This heterogeneity may limit the sensitivity of albumin-centered screening strategies and underscores the need for complementary biomarkers that capture renal injury beyond glomerular albumin leakage, particularly those reflecting tubular injury and dysfunction (10, 11).

Against this background, the present multicenter retrospective study evaluated the diagnostic performance of routinely available urinary glomerular and tubular biomarkers for identifying DN in hospitalized patients with type 2 diabetes mellitus. In addition to comparing individual biomarkers, we examined their stage-wise behavior across KDIGO eGFR categories and assessed whether combining tubular and glomerular markers could improve diagnostic discrimination beyond single-marker approaches. By focusing on clinically accessible biomarkers that are already used in routine practice, this study aimed to provide evidence with direct translational relevance for real-world risk stratification. We hypothesized that a combined tubular–glomerular biomarker strategy would improve diagnostic accuracy for DN compared with individual biomarkers alone.

## 2 Methods

### 2.1 Study design

This multicenter retrospective observational study enrolled hospitalized patients with type 2 diabetes mellitus (T2DM) who were managed at Henan Provincial Hospital of Traditional Chinese Medicine, The First Affiliated Hospital of Henan University of Chinese Medicine, and The First Affiliated Hospital of Zhengzhou University between January 2022 and December 2025. Patients were categorized according to the presence or absence of DN, and renal impairment severity was further stratified exclusively by eGFR into predefined kidney function stages to evaluate stage-dependent changes. Inclusion criteria were: (i) age ≥18 years; (ii) confirmed diagnosis of T2DM; (iii) inpatient admission during the study period; (iv) availability of serum creatinine data sufficient to calculate eGFR and assign eGFR stage; and (v) at least one eligible tubular and/or glomerular biomarker measurement within the prespecified assessment window. Exclusion criteria were: (i) non-diabetic primary kidney diseases or secondary nephropathies (e.g., glomerulonephritis, lupus nephritis, vasculitis); (ii) acute kidney injury at admission or during the biomarker assessment window; (iii) end-stage kidney disease requiring maintenance dialysis or prior kidney transplantation; (iv) active malignancy, severe systemic infection/sepsis, decompensated liver failure, or other critical illnesses likely to substantially confound biomarker concentrations; (v) pregnancy; and (vi) missing key data precluding eGFR calculation, stage allocation, or principal analyses. The study was reviewed and approved by the hospital ethics committee. All procedures were conducted in accordance with relevant guidelines and the Declaration of Helsinki. Informed consent was obtained from all participants. Data were anonymized prior to analysis to ensure confidentiality and protect participant privacy.

### 2.2 Diagnostic definitions and staging criteria

T2DM was ascertained from the electronic medical record based on documented admission and discharge diagnoses or a verified prior diagnosis in historical medical records. The diagnosis of T2DM was determined in accordance with widely accepted clinical diagnostic criteria and contemporary guideline recommendations for diabetes mellitus, integrating glycemic thresholds with clinical phenotyping consistent with T2DM. When discrepancies existed among records, the discharge diagnosis and corroborated prior diagnostic documentation were prioritized. All case classifications were independently reviewed by two investigators, with disagreements resolved by consensus.

For operational classification in this retrospective study, DN was assigned when patients with T2DM had a clinician-documented diagnosis of DN/diabetic kidney disease in the medical record or discharge diagnosis, together with evidence of diabetic kidney involvement and no more plausible alternative cause of kidney disease after review of the available clinical data. Because of the retrospective design and incomplete longitudinal availability of repeat outpatient measurements, persistent albuminuria was not required in every case for DN classification. Patients with reduced eGFR but without documented albuminuria were classified as DN only when the clinical record supported diabetic kidney disease and other primary or secondary renal diseases had been excluded on the basis of available history, laboratory findings, imaging, and, when available, pathology. In the absence of albuminuria and without sufficient clinical evidence supporting diabetic kidney disease, such patients were not classified as DN. Diabetic microvascular complications, including diabetic retinopathy when documented, were treated as supportive but non-mandatory evidence for DN classification.

Kidney function was quantified using eGFR, expressed as mL/min/1.73 m². eGFR was calculated from serum creatinine, age, and sex using the 2021 Chronic Kidney Disease Epidemiology Collaboration (CKD-EPI) creatinine equation. The same equation was applied uniformly across all three participating centers to ensure inter-center consistency. For patients with repeated serum creatinine measurements during hospitalization, the value closest in time to the biomarker assessment was used to compute eGFR to ensure temporal alignment between staging and biomarker sampling. When multiple eligible measurements were available within the prespecified window, *a priori* rules were applied consistently to select the measurement used for staging.

Participants were stratified according to the KDIGO eGFR categories to characterize kidney injury severity and to support stage-wise comparisons and trend analyses. eGFR stages were defined as G1 (≥90 mL/min/1.73 m²), G2 (60–89 mL/min/1.73 m²), G3 (30–59 mL/min/1.73 m²), G4 (15–29 mL/min/1.73 m²), and G5 (<15 mL/min/1.73 m²). This KDIGO-based stratification was applied to compare the distributions of tubular and glomerular biomarkers across stages, assess dose–response relationships with declining eGFR, and perform stage-specific between-group comparisons between the DN and non-DN groups, thereby enabling an evaluation of biomarker performance across progressive kidney dysfunction.

### 2.3 Data collection

Demographic characteristics (age, sex, and body mass index), diabetes-related information (duration of type 2 diabetes, glycated hemoglobin, and fasting plasma glucose), comorbidities (hypertension and coronary artery disease), and medication exposures during hospitalization or at discharge were systematically collected using prespecified case-report forms. Medication exposures included antihyperglycemic therapy (insulin, oral hypoglycemic agents, sodium–glucose cotransporter-2 inhibitors, and glucagon-like peptide-1 receptor agonists), renin–angiotensin system blockers, and statins. Laboratory data were retrieved from the hospital laboratory information systems and were matched to the index hospitalization. Routine biochemical indices included serum creatinine, blood urea nitrogen, uric acid, electrolytes (sodium, potassium, chloride), lipid profile (total cholesterol, low-density lipoprotein cholesterol, high-density lipoprotein cholesterol, triglycerides), and C-reactive protein. Renal function was quantified by estimated glomerular filtration rate, calculated from serum creatinine, age, and sex using the prespecified equation, and was used to assign eGFR categories (G1–G5). Urine testing data included urine dipstick protein, urine albumin-to-creatinine ratio, and urinary biomarkers representing glomerular permeability (urinary albumin, urinary transferrin, urinary immunoglobulin G) and tubular injury or proximal tubular dysfunction (urinary N-acetyl-β-D-glucosaminidase, urinary β2-microglobulin, urinary α1-microglobulin, urinary retinol-binding protein). When multiple laboratory measurements were available during the index admission, the value closest in time to the urine biomarker assessment was selected; if urine biomarkers were measured more than once, the first available measurement meeting quality requirements was used for the primary analysis. Data completeness and plausibility were reviewed prior to analysis; key variables required for cohort definition, eGFR calculation, and biomarker evaluation were verified, and records with missing core measurements were excluded according to prespecified criteria.

### 2.4 Data extraction workflow and quality control

Two investigators independently extracted variables from the electronic medical record and laboratory information systems and then cross-checked all entries. Discrepancies were resolved by review of the source records, and a third investigator adjudicated unresolved disagreements. To harmonize data across the three participating hospitals, units and coding rules were standardized before analysis. A fixed testing window was defined *a priori*. For routine laboratory indices, the primary value was the first measurement obtained within 24–48 hours after admission during the index hospitalization. Urinary tubular and glomerular biomarkers were linked to the index admission; when biomarker sampling occurred outside 48 hours, the earliest available biomarker result obtained during hospitalization was used, and the corresponding blood and routine urine test results closest in time to that biomarker measurement were selected. For repeated measurements within the prespecified window, the earliest value was used for the primary analysis; if no value was available within the window, the closest value within 72 hours was selected. Urinary biomarkers were measured across the three centers using the same manufacturer-matched reagents or comparable validated assay systems on routine automated platforms, with harmonized specimen handling, calibration, and analytical procedures. Urinary biomarkers were measured across the three centers using harmonized analytical procedures on routine automated platforms. Urinary albumin, urinary transferrin, urinary immunoglobulin G, urinary β2-microglobulin, and urinary retinol-binding protein were measured mainly by immunoturbidimetric assays; urinary α1-microglobulin was measured by immunonephelometric or immunoturbidimetric assay depending on platform configuration; urinary N-acetyl-β-D-glucosaminidase was measured by an enzymatic colorimetric/spectrophotometric assay. Urine creatinine was measured enzymatically, and UACR was calculated accordingly. All laboratories performed routine internal quality control and participated in external quality assessment or proficiency testing when available. Within-assay and between-assay coefficients of variation were summarized to characterize assay precision, and inter-laboratory consistency was assessed descriptively on the basis of assay harmonization and quality-control performance. Additional data quality-control procedures included unit harmonization, range and consistency checks, and verification of outliers against source laboratory reports. Estimated glomerular filtration rate was recalculated programmatically for all participants using the prespecified 2021 CKD-EPI creatinine equation and independently re-validated to ensure consistency across centers. Missingness was assessed for each variable. Records lacking core elements required for cohort definition, eGFR staging, or primary biomarker evaluation were excluded; remaining analyses primarily used complete-case data, with prespecified sensitivity analyses restricted to records with complete key variables. Several tubular biomarkers were routinely reported as ratios to urinary creatinine, which partially mitigated the influence of urine concentration on measured values.

### 2.5 Statistical analysis

All statistical analyses were performed using IBM SPSS Statistics, version 28.0 (IBM Corp., Armonk, NY, USA). Continuous variables were assessed for normality using the Shapiro–Wilk test and are presented as mean ± standard deviation for approximately normally distributed data or as median with interquartile range for skewed data. Categorical variables are summarized as counts and percentages. Between-group comparisons (DN versus controls without nephropathy) were conducted using the independent-samples t test for normally distributed continuous variables and the Mann–Whitney U test for non-normally distributed variables; categorical variables were compared using the chi-square test or Fisher’s exact test, as appropriate. Comparisons across ordered eGFR stages (G1–G5) were performed using one-way analysis of variance or the Kruskal–Wallis test, as appropriate. Medication exposure variables were summarized as counts and percentages and compared between groups using the chi-square test or Fisher’s exact test. Diagnostic performance was evaluated using receiver operating characteristic curve analysis, with the area under the curve (AUC) and 95% confidence intervals reported. Optimal cutoffs were determined by the Youden index, with corresponding sensitivity, specificity, positive predictive value, and negative predictive value. Multivariable logistic regression was used to develop combined diagnostic models integrating tubular and glomerular biomarkers, with optional inclusion of clinical covariates. Comparisons between correlated AUCs were conducted using the DeLong test. Model calibration was assessed using the Hosmer–Lemeshow goodness-of-fit test and the Brier score. Fully adjusted sensitivity analyses were performed using multivariable logistic regression with DN status as the dependent variable. A covariate-only model and models additionally including UACR, urinary β2-microglobulin, urinary N-acetyl-β-D-glucosaminidase, or the combined biomarker score were fitted after adjustment for age, sex, body mass index, diabetes duration, HbA1c, hypertension, renin–angiotensin system blocker use, sodium–glucose cotransporter-2 inhibitor use, glucagon-like peptide-1 receptor agonist use, statin use, and insulin use. Biomarkers were log-transformed and standardized as appropriate, and adjusted odds ratios were reported per 1-standard-deviation increase. Model discrimination, calibration, and incremental discrimination versus the covariate-only model were evaluated using the AUC, Hosmer–Lemeshow test, Brier score, and DeLong test, respectively. Multicollinearity among predictors in the primary combined biomarker models was assessed using variance inflation factors (VIFs), with VIF values >5 considered indicative of potentially important multicollinearity. Internal validation of the primary combined biomarker models was performed using 1,000 bootstrap resamples. Optimism was estimated as the mean difference in model performance between the bootstrap and original samples, and optimism-corrected estimates were obtained by subtracting this value from the apparent performance. Validation metrics included the optimism-corrected AUC, Brier score, and calibration slope. Prespecified subgroup analyses evaluated model performance across clinically relevant strata, and sensitivity analyses were performed by excluding admissions with infection or sepsis and those with extreme serum creatinine values. Stage-specific biomarker P values were adjusted for multiple comparisons using the Benjamini–Hochberg false discovery rate procedure, with statistical significance defined as an FDR-adjusted P value <0.05. All tests were two-sided, and a P value <0.05 was considered statistically significant unless otherwise specified.

## 3 Results

### 3.1 Study population and participant flow

**Figure 1:**
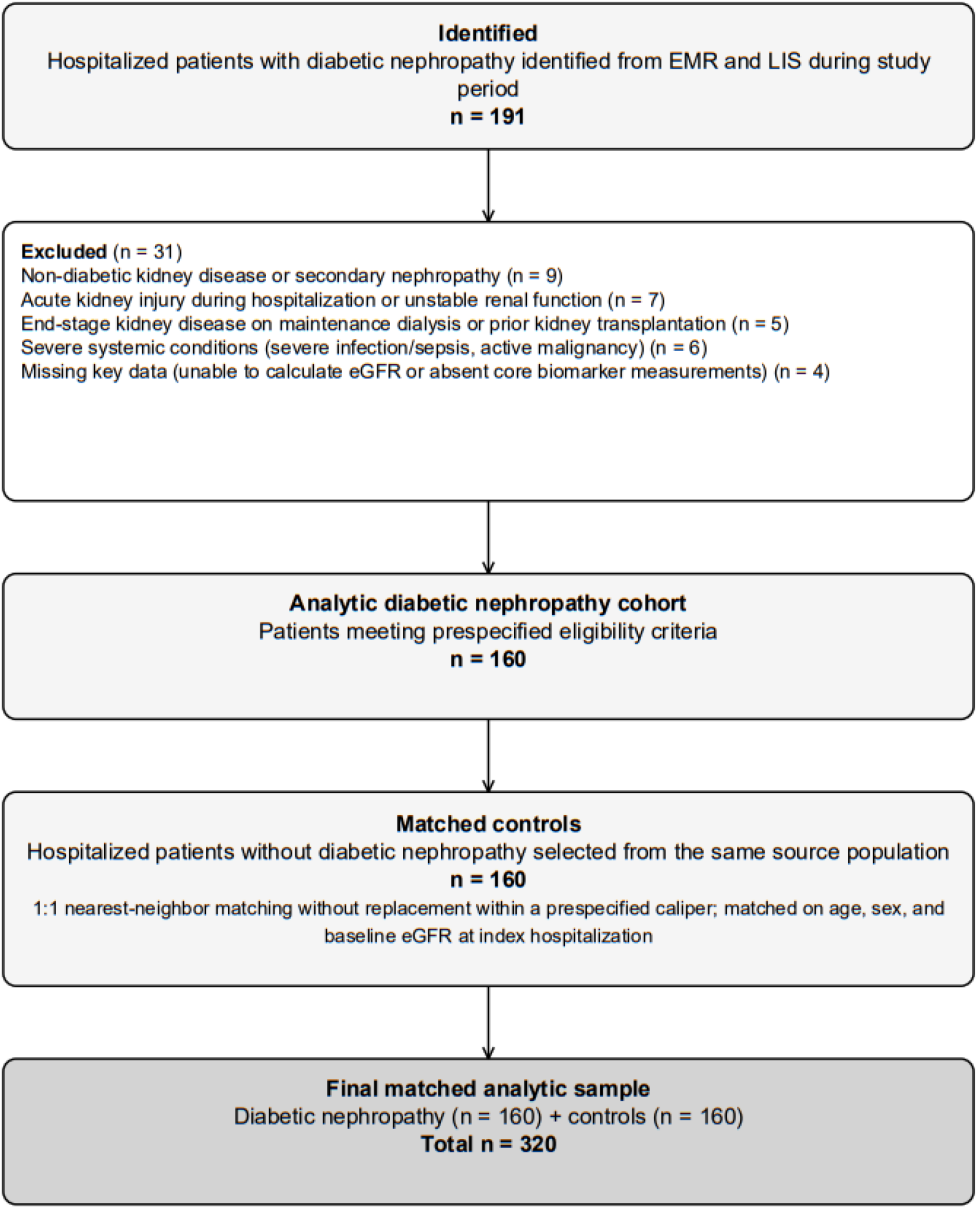
Participant selection and matching flow diagram.

A total of 191 hospitalized patients with DN were initially identified from the electronic medical record and laboratory information systems during the study period. After application of the prespecified eligibility criteria, 31 patients were excluded, leaving 160 patients with DN in the analytic cohort. A control group of 160 hospitalized patients with T2DM but without DN was then selected from the same source population and matched to the DN group in a 1:1 ratio by age and sex using nearest-neighbor matching without replacement and within a prespecified caliper. The final study sample therefore comprised 320 participants. The 31 exclusions were due to non-diabetic kidney disease or secondary nephropathy (n = 9), acute kidney injury during hospitalization or unstable renal function precluding reliable staging (n = 7), end-stage kidney disease receiving maintenance dialysis or prior kidney transplantation (n = 5), severe systemic conditions likely to substantially influence biomarker profiles, including severe infection or sepsis and active malignancy (n = 6), and missing key data preventing eGFR calculation or absence of core biomarker measurements (n = 4) (**Figure 1**).

### 3.2 Baseline characteristics

Baseline demographic features were comparable between groups, with no significant differences in age, sex distribution, or BMI. Compared with controls without nephropathy, patients in the DN group had a longer diabetes duration and higher glycemic indices, including HbA1c and fasting plasma glucose. The DN group also showed a higher prevalence of hypertension and a higher proportion of insulin use. In addition, patients with DN more frequently received renin–angiotensin system blockers and statins, reflecting differential cardiovascular and renal risk management patterns between groups. Use of sodium–glucose cotransporter-2 inhibitors and glucagon-like peptide-1 receptor agonists was relatively low in both groups and did not differ significantly between groups. Renal function and urine indices differed substantially between groups, with higher serum creatinine and blood urea nitrogen, lower eGFR, and markedly higher levels of albuminuria and proteinuria in the DN group (**Table 1**). Assay precision for the primary urinary biomarkers was acceptable overall, with within-assay and between-assay coefficients of variation remaining within prespecified laboratory quality-control ranges. In addition, no material inter-laboratory inconsistency was identified on review of harmonized assay procedures and quality-control performance across the three participating centers (**Supplementary Table 1**).

**Table 1:** Baseline demographic and clinical characteristics in the DN group and matched T2DM controls without nephropathy.

Variable	DN (n = 160)	Controls without nephropathy (n = 160)	Test statistic	P value
Age, years	61.20 ± 9.80	60.70 ± 10.10	t = 0.45	0.653
Male sex, n (%)	98 (61.3)	96 (60.0)	χ² = 0.05	0.819
BMI, kg/m²	25.10 ± 3.60	24.60 ± 3.40	t = 1.28	0.202
Diabetes duration, years	12.30 ± 6.10	8.10 ± 5.40	t = 6.52	<0.001
HbA1c, %	8.90 ± 1.60	8.20 ± 1.50	t = 4.04	<0.001
Fasting plasma glucose, mmol/L	9.80 ± 3.10	9.10 ± 2.90	t = 2.09	0.038
Hypertension, n (%)	118 (73.8)	96 (60.0)	χ² = 6.83	0.009
Coronary artery disease, n (%)	44 (27.5)	30 (18.8)	χ² = 3.45	0.063
Current smoker, n (%)	38 (23.8)	36 (22.5)	χ² = 0.07	0.791
Insulin use, n (%)	92 (57.5)	74 (46.2)	χ² = 4.06	0.044
Oral hypoglycemic agents, n (%)	134 (83.8)	142 (88.8)	χ² = 1.69	0.194
SGLT2 inhibitor use, n (%)	22 (13.8)	28 (17.5)	χ² = 0.59	0.441
GLP-1 receptor agonist use, n (%)	12 (7.5)	16 (10.0)	χ² = 0.35	0.553
RAS blocker use, n (%)	104 (65.0)	62 (38.8)	χ² = 22.08	<0.001
Statin use, n (%)	96 (60.0)	78 (48.8)	χ² = 4.08	0.043
Serum creatinine, µmol/L	132.00 ± 58.00	78.00 ± 18.00	t = 11.25	<0.001
Blood urea nitrogen, mmol/L	9.20 ± 4.10	5.80 ± 1.70	t = 9.69	<0.001
eGFR, mL/min/1.73 m²	53.00 ± 23.00	92.00 ± 16.00	t = −17.61	<0.001
Urine albumin-to-creatinine ratio category, n (%)			χ² = 191.89	<0.001
<30 mg/g	18 (11.2)	140 (87.5)	—	—
30–300 mg/g	82 (51.2)	20 (12.5)	—	—
>300 mg/g	60 (37.5)	0 (0.0)	—	—
Proteinuria (dipstick ≥1+), n (%)	96 (60.0)	12 (7.5)	χ² = 98.62	<0.001

DN, diabetic nephropathy; T2DM, type 2 diabetes mellitus; BMI, body mass index; HbA1c, glycated hemoglobin A1c; RAS, renin–angiotensin system; eGFR, estimated glomerular filtration rate; UACR, urine albumin-to-creatinine ratio.

### 3.3 Stage-wise distribution of clinical and laboratory characteristics across eGFR categories (G1–G5)

Serum creatinine and blood urea nitrogen increased progressively from G1 to G5, with strong evidence of between-stage differences (both overall P < 0.001) and statistically significant monotonic trends across worsening eGFR categories (both P for trend < 0.001). Uric acid levels also differed across stages (P < 0.001) and showed a monotonic increase with advancing stage (P for trend < 0.001), consistent with greater impairment of renal urate handling in more advanced kidney dysfunction. Electrolytes did not demonstrate robust stage-dependent differences. Sodium, potassium, and chloride were not significantly different across eGFR categories in the overall comparison, and trend testing did not support consistent monotonic changes across stages. Similarly, lipid parameters (total cholesterol, LDL-C, HDL-C) and triglycerides did not differ significantly by eGFR category, and no monotonic trends were observed. Inflammatory burden increased with advancing kidney dysfunction. C-reactive protein rose across eGFR categories, with significant between-stage heterogeneity (P < 0.001) and a significant positive monotonic trend (P for trend < 0.001) (**Table 2**).

**Table 2:** Clinical and laboratory characteristics across KDIGO eGFR categories (G1–G5) in the overall cohort.

Variable	G1 (n = 150)	G2 (n = 90)	G3 (n = 50)	G4 (n = 20)	G5 (n = 10)	Overall test statistic	P value	P for trend
Serum creatinine, µmol/L	81.15 ± 12.80	94.73 ± 16.56	138.98 ± 35.67	238.86 ± 41.06	404.60 ± 85.03	F = 522.81	<0.001	<0.001
Blood urea nitrogen, mmol/L	5.59 ± 1.09	6.69 ± 1.23	10.46 ± 3.45	17.89 ± 3.94	22.64 ± 7.44	F = 252.51	<0.001	<0.001
Uric acid, µmol/L	342.31 ± 70.64	348.12 ± 70.94	372.57 ± 66.30	397.46 ± 79.40	413.50 ± 72.37	F = 5.70	<0.001	<0.001
Sodium, mmol/L	139.97 ± 2.59	140.31 ± 2.69	140.13 ± 3.10	141.05 ± 2.24	140.40 ± 2.14	F = 0.83	0.507	0.150
Potassium, mmol/L	4.21 ± 0.34	4.32 ± 0.43	4.24 ± 0.38	4.29 ± 0.47	4.06 ± 0.41	F = 1.91	0.108	0.528
Chloride, mmol/L	102.12 ± 3.49	101.70 ± 3.70	101.81 ± 3.82	101.94 ± 2.99	101.27 ± 3.53	F = 0.30	0.881	0.445
Total cholesterol, mmol/L	4.61 ± 0.76	4.42 ± 0.84	4.59 ± 0.89	4.44 ± 0.79	4.52 ± 0.71	F = 0.89	0.472	0.153
LDL-C, mmol/L	2.55 ± 0.63	2.63 ± 0.69	2.50 ± 0.82	2.63 ± 0.61	2.39 ± 0.60	F = 0.55	0.698	0.832
HDL-C, mmol/L	1.11 ± 0.26	1.15 ± 0.23	1.14 ± 0.25	1.12 ± 0.22	1.17 ± 0.23	F = 0.57	0.683	0.256
Triglycerides, mmol/L	1.82 (1.07, 2.72)	1.41 (1.06, 2.75)	2.12 (1.23, 3.13)	1.69 (1.24, 2.22)	1.67 (1.39, 1.97)	H = 2.32	0.678	0.747
C-reactive protein, mg/L	1.88 (1.18, 3.00)	2.12 (1.25, 3.71)	3.69 (2.03, 5.36)	4.09 (3.07, 5.16)	4.68 (2.94, 6.79)	H = 34.56	<0.001	<0.001

Overall, between-stage differences were assessed using one-way ANOVA (F) or the Kruskal–Wallis test (H), as appropriate. Trend across ordered eGFR categories (G1–G5) was evaluated using a prespecified Spearman rank trend test.

KDIGO, Kidney Disease: Improving Global Outcomes; LDL-C, low-density lipoprotein cholesterol; HDL-C, high-density lipoprotein cholesterol.

### 3.4 Glomerular biomarkers—between-group differences and stage-wise trends across eGFR categories

In the overall comparison, patients with DN showed markedly elevated UACR, urinary albumin, urinary transferrin, and urinary IgG compared with controls without nephropathy (all P < 0.001). This separation was already evident in preserved kidney function. Within G1, median UACR was 61.44 mg/g in the DN group versus 11.86 mg/g in controls (Z = 9.10, P < 0.001; FDR-adjusted P = 0.0013), and similarly significant differences were observed for urinary albumin (46.86 vs 7.88 mg/L; Z = 10.12, P < 0.001; FDR-adjusted P = 0.0013), urinary transferrin (1.94 vs 0.73 mg/L; Z = 7.43, P < 0.001; FDR-adjusted P = 0.0013), and urinary IgG (3.57 vs 0.96 mg/L; Z = 8.16, P < 0.001; FDR-adjusted P = 0.0013). These findings indicate that albuminuria-related measures and glomerular permeability markers can distinguish DN from controls even before substantial loss of eGFR. Across eGFR stages, glomerular biomarker levels in the DN group increased progressively with worsening kidney function, with significant monotonic trends for UACR (ρ = 0.59, P < 0.001), urinary albumin (ρ = 0.59, P < 0.001), urinary transferrin (ρ = 0.65, P < 0.001), and urinary IgG (ρ = 0.51, P < 0.001). In contrast, biomarker levels in controls remained low overall, and no significant monotonic trends were observed (all trend P > 0.05).

Stage-specific comparisons further showed that, from G1 through G5, patients with DN consistently had significantly higher levels of UACR, urinary albumin, urinary transferrin, and urinary IgG than controls. All stage-specific differences remained statistically significant after Benjamini–Hochberg false discovery rate correction, with FDR-adjusted P values ranging from 0.0013 to 0.0123 for UACR, 0.0013 to 0.0095 for urinary albumin, 0.0013 to 0.0095 for urinary transferrin, and 0.0013 to 0.0095 for urinary IgG. Notably, urinary transferrin and urinary IgG demonstrated clear discrimination in early-stage disease and continued to increase across worsening eGFR categories in DN, suggesting sensitivity to progressive impairment of glomerular barrier integrity (**Table 3**).

**Table 3:** Glomerular biomarkers: between-group differences (DN vs controls without nephropathy) and eGFR stage–wise patterns (G1–G5).

Biomarker/eGFR stage	DN (n = 160), median (IQR)	Controls (n = 160), median (IQR)	Test statistic	P value	FDR-adjusted P value	Trend within DN (Spearman ρ, P)	Trend within controls (Spearman ρ, P)
UACR (mg/g) — Overall	96.67 (57.04, 214.08)	11.86 (7.12, 22.43)	Z = 14.14	<0.001	—	0.59, <0.001	0.05, 0.508
G1	61.44 (40.27, 101.10)	11.86 (7.12, 22.43)	Z = 9.10	<0.001	0.0013	—	—
G2	128.08 (77.83, 207.63)	11.86 (7.12, 22.43)	Z = 7.93	<0.001	0.0013	—	—
G3	281.76 (136.71, 549.41)	11.86 (7.12, 22.43)	Z = 8.35	<0.001	0.0013	—	—
G4	618.39 (317.20, 1292.37)	11.86 (7.12, 22.43)	Z = 5.24	<0.001	0.0013	—	—
G5	972.34 (490.33, 1907.06)	11.86 (7.12, 22.43)	Z = 2.51	0.012	0.0123	—	—
UAlb (mg/L) — Overall	78.08 (42.85, 170.76)	7.88 (4.77, 12.15)	Z = 14.73	<0.001	—	0.59, <0.001	0.02, 0.801
G1	46.86 (31.05, 78.17)	7.88 (4.77, 12.15)	Z = 10.12	<0.001	0.0013	—	—
G2	75.17 (43.88, 131.67)	7.88 (4.77, 12.15)	Z = 7.87	<0.001	0.0013	—	—
G3	162.94 (91.41, 331.87)	7.88 (4.77, 12.15)	Z = 8.53	<0.001	0.0013	—	—
G4	286.27 (164.52, 567.92)	7.88 (4.77, 12.15)	Z = 5.24	<0.001	0.0013	—	—
G5	485.66 (262.57, 762.05)	7.88 (4.77, 12.15)	Z = 2.61	0.009	0.0095	—	—
uTRF (mg/L) — Overall	3.82 (2.02, 7.19)	0.73 (0.39, 1.21)	Z = 13.26	<0.001	—	0.65, <0.001	0.08, 0.299
G1	1.94 (1.19, 2.99)	0.73 (0.39, 1.21)	Z = 7.43	<0.001	0.0013	—	—
G2	3.86 (2.30, 5.22)	0.73 (0.39, 1.21)	Z = 6.85	<0.001	0.0013	—	—
G3	6.69 (3.88, 10.92)	0.73 (0.39, 1.21)	Z = 7.86	<0.001	0.0013	—	—
G4	10.44 (6.19, 16.27)	0.73 (0.39, 1.21)	Z = 5.24	<0.001	0.0013	—	—
G5	16.65 (9.43, 21.48)	0.73 (0.39, 1.21)	Z = 2.61	0.009	0.0095	—	—
uIgG (mg/L) — Overall	6.03 (3.14, 11.52)	0.96 (0.50, 1.72)	Z = 12.36	<0.001	—	0.51, <0.001	−0.11, 0.149
G1	3.57 (2.28, 6.72)	0.96 (0.50, 1.72)	Z = 8.16	<0.001	0.0013	—	—
G2	5.45 (3.12, 8.60)	0.96 (0.50, 1.72)	Z = 6.92	<0.001	0.0013	—	—
G3	10.49 (5.74, 18.47)	0.96 (0.50, 1.72)	Z = 7.62	<0.001	0.0013	—	—
G4	17.93 (10.28, 31.35)	0.96 (0.50, 1.72)	Z = 5.24	<0.001	0.0013	—	—
G5	23.12 (13.39, 41.06)	0.96 (0.50, 1.72)	Z = 2.61	0.009	0.0095	—	—

DN, diabetic nephropathy; eGFR, estimated glomerular filtration rate; IQR, interquartile range; UACR, urine albumin-to-creatinine ratio; UAlb, urinary albumin; uTRF, urinary transferrin; uIgG, urinary immunoglobulin G.

### 3.5 Clinically common tubular biomarkers—between-group differences and eGFR stage–wise trends

In the overall comparison, urinary NAG, urinary β2-microglobulin, urinary α1-microglobulin, and urinary retinol-binding protein were significantly elevated in patients with DN compared with controls without nephropathy (all P < 0.001), supporting a higher burden of tubular injury and impaired proximal tubular reabsorption in DN. This separation was already evident in preserved kidney function. Within G1, DN patients had higher urinary NAG (2.17 vs 1.03 U/g creatinine; Z = 7.87, P < 0.001; FDR-adjusted P = 0.0013), urinary β2-microglobulin (0.90 vs 0.37 mg/g creatinine; Z = 6.66, P < 0.001; FDR-adjusted P = 0.0013), urinary α1-microglobulin (1.37 vs 0.81 mg/g creatinine; Z = 6.46, P < 0.001; FDR-adjusted P = 0.0013), and urinary retinol-binding protein (0.83 vs 0.45 mg/g creatinine; Z = 6.11, P < 0.001; FDR-adjusted P = 0.0013). These findings indicate that commonly used tubular biomarkers can distinguish DN from controls even before substantial loss of eGFR. Across eGFR stages, tubular biomarker levels in the DN group increased significantly with worsening kidney function, as shown by monotonic trend analyses for urinary NAG (ρ = 0.59, P < 0.001), urinary β2-microglobulin (ρ = 0.67, P < 0.001), urinary α1-microglobulin (ρ = 0.43, P < 0.001), and urinary retinol-binding protein (ρ = 0.55, P < 0.001). In contrast, biomarker levels in controls remained low overall, and no significant monotonic trends were observed (all trend P > 0.05).

Stage-specific comparisons further showed that, from G1 through G5, DN patients consistently had significantly higher levels of urinary NAG, urinary β2-microglobulin, urinary α1-microglobulin, and urinary retinol-binding protein than controls. All stage-specific differences remained statistically significant after Benjamini–Hochberg false discovery rate correction, with FDR-adjusted P values ranging from 0.0013 to 0.0095 for urinary NAG, 0.0013 to 0.0095 for urinary β2-microglobulin, 0.0013 to 0.0095 for urinary α1-microglobulin, and 0.0013 to 0.0160 for urinary retinol-binding protein. Overall, urinary NAG and low-molecular-weight protein markers, particularly urinary β2-microglobulin, demonstrated both early-stage discrimination and pronounced stage-wise gradients, supporting their utility as clinically practical tubular indicators across the spectrum of kidney dysfunction severity (**Table 4**).

**Table 4:** Clinically common tubular biomarkers: between-group differences (DN vs controls without nephropathy) and eGFR stage–wise patterns (G1–G5).

Biomarker/eGFR stage	DN, median (IQR)	Controls, median (IQR)	Test statistic	P value	FDR-adjusted P value	Trend within DN (ρ, P)	Trend within controls (ρ, P)
Urinary NAG, U/g creatinine — Overall	3.05 (2.14, 4.64)	1.03 (0.68, 1.56)	Z = 13.63	<0.001	—	0.59, <0.001	0.02, 0.755
G1	2.17 (1.60, 3.02)	1.03 (0.68, 1.56)	Z = 7.87	<0.001	0.0013	—	—
G2	2.86 (2.07, 3.94)	1.03 (0.68, 1.56)	Z = 7.41	<0.001	0.0013	—	—
G3	4.32 (3.08, 6.03)	1.03 (0.68, 1.56)	Z = 5.87	<0.001	0.0013	—	—
G4	5.79 (4.05, 8.76)	1.03 (0.68, 1.56)	Z = 3.78	<0.001	0.0013	—	—
G5	6.54 (5.23, 9.15)	1.03 (0.68, 1.56)	Z = 2.61	0.009	0.0095	—	—
Urinary β2-microglobulin, mg/g creatinine — Overall	1.28 (0.86, 2.76)	0.37 (0.24, 0.67)	Z = 11.94	<0.001	—	0.67, <0.001	−0.03, 0.745
G1	0.90 (0.64, 1.34)	0.37 (0.24, 0.67)	Z = 6.66	<0.001	0.0013	—	—
G2	1.12 (0.83, 1.84)	0.37 (0.24, 0.67)	Z = 7.30	<0.001	0.0013	—	—
G3	2.28 (1.37, 3.64)	0.37 (0.24, 0.67)	Z = 5.99	<0.001	0.0013	—	—
G4	4.94 (3.01, 7.55)	0.37 (0.24, 0.67)	Z = 3.78	<0.001	0.0013	—	—
G5	7.90 (5.94, 10.40)	0.37 (0.24, 0.67)	Z = 2.61	0.009	0.0095	—	—
Urinary α1-microglobulin, mg/g creatinine — Overall	2.03 (1.40, 3.42)	0.81 (0.54, 1.35)	Z = 11.71	<0.001	—	0.43, <0.001	0.05, 0.530
G1	1.37 (1.05, 1.92)	0.81 (0.54, 1.35)	Z = 6.46	<0.001	0.0013	—	—
G2	1.93 (1.41, 2.78)	0.81 (0.54, 1.35)	Z = 7.19	<0.001	0.0013	—	—
G3	3.29 (2.29, 4.90)	0.81 (0.54, 1.35)	Z = 4.59	<0.001	0.0013	—	—
G4	3.44 (2.53, 4.77)	0.81 (0.54, 1.35)	Z = 3.48	0.001	0.0013	—	—
G5	4.12 (3.44, 5.36)	0.81 (0.54, 1.35)	Z = 2.61	0.009	0.0095	—	—
Urinary retinol-binding protein, mg/g creatinine — Overall	1.14 (0.79, 1.84)	0.45 (0.28, 0.79)	Z = 10.76	<0.001	—	0.55, <0.001	−0.02, 0.836
G1	0.83 (0.63, 1.10)	0.45 (0.28, 0.79)	Z = 6.11	<0.001	0.0013	—	—
G2	1.60 (1.09, 2.26)	0.45 (0.28, 0.79)	Z = 7.75	<0.001	0.0013	—	—
G3	2.05 (1.32, 3.04)	0.45 (0.28, 0.79)	Z = 5.23	<0.001	0.0013	—	—
G4	1.86 (1.14, 2.38)	0.45 (0.28, 0.79)	Z = 2.95	0.003	0.0038	—	—
G5	1.02 (0.86, 1.38)	0.45 (0.28, 0.79)	Z = 2.40	0.016	0.016	—	—

DN, diabetic nephropathy; eGFR, estimated glomerular filtration rate; IQR, interquartile range; NAG, N-acetyl-β-D-glucosaminidase.

### 3.6 Diagnostic utility of single biomarkers for DN identification and stage discrimination

**Figure 2:**
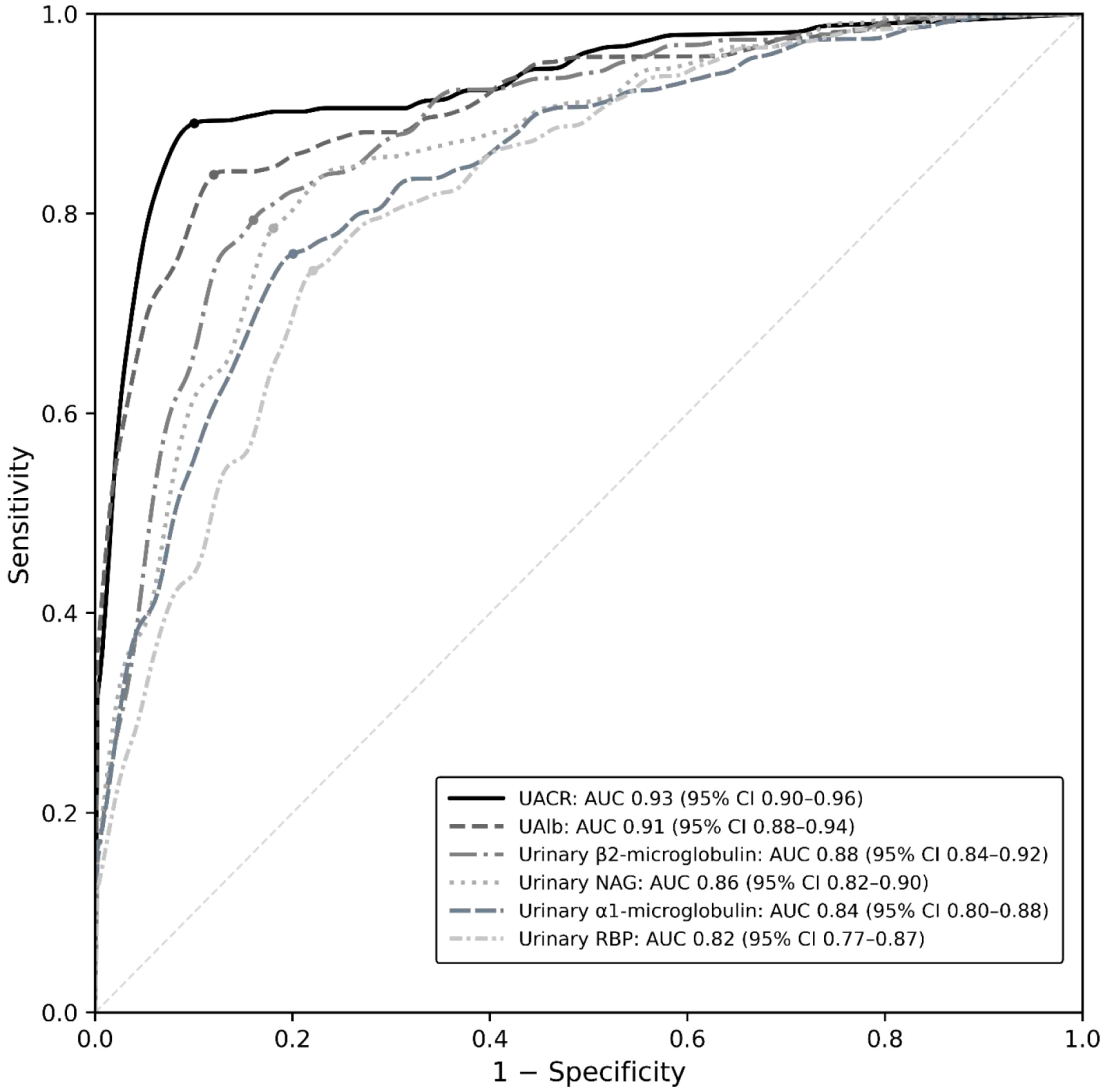
Receiver operating characteristic curves of single urinary biomarkers for identifying diabetic nephropathy, showing that UACR achieved the highest discriminative performance, followed by urinary albumin, urinary β2-microglobulin, urinary N-acetyl-β-D-glucosaminidase, urinary α1-microglobulin, and urinary retinol-binding protein.

Single-biomarker ROC analyses showed that glomerular markers provided the highest discrimination for DN status. UACR yielded the largest AUC (0.93, 95% CI 0.90–0.96; P < 0.001), with an optimal cutoff of 28 mg/g producing sensitivity of 89.0% and specificity of 90.0%. Urinary albumin demonstrated similarly strong diagnostic performance (AUC 0.91, 95% CI 0.88–0.94; P < 0.001), with sensitivity of 86.0% and specificity of 88.0% at the Youden-derived threshold. Among clinically common tubular markers, urinary β2-microglobulin showed the best DN discrimination (AUC 0.88, 95% CI 0.84–0.92; P < 0.001), followed by urinary NAG (AUC 0.86, 95% CI 0.82–0.90; P < 0.001). Urinary α1-microglobulin and urinary RBP provided moderate discrimination (AUC 0.84 and 0.82, respectively; both P < 0.001). Using the study prevalence of DN (50%), PPV and NPV were highest for UACR (89.9% and 89.1%) and urinary albumin (87.8% and 86.3%), consistent with their superior AUCs (**Table 5**; **Figure 2**).

**Table 5:** Diagnostic performance of single biomarkers for identifying diabetic nephropathy (DN).

Biomarker	AUC (95% CI)	P for AUC > 0.50	Optimal cutoff (Youden)	Sensitivity, %	Specificity, %	PPV, %	NPV, %
UACR, mg/g	0.93 (0.90–0.96)	<0.001	28 mg/g	89.0	90.0	89.9	89.1
Urinary albumin (UAlb), mg/L	0.91 (0.88–0.94)	<0.001	16 mg/L	86.0	88.0	87.8	86.3
Urinary β2-microglobulin, mg/g creatinine	0.88 (0.84–0.92)	<0.001	0.80 mg/g	82.0	84.0	83.7	82.4
Urinary NAG, U/g creatinine	0.86 (0.82–0.90)	<0.001	1.80 U/g	80.0	82.0	81.6	80.4
Urinary α1-microglobulin, mg/g creatinine	0.84 (0.80–0.88)	<0.001	1.15 mg/g	78.0	80.0	79.6	78.4
Urinary RBP, mg/g creatinine	0.82 (0.77–0.87)	<0.001	0.70 mg/g	75.0	78.0	77.3	75.7

AUC, area under the receiver operating characteristic curve; DN, diabetic nephropathy; NAG, N-acetyl-β-D-glucosaminidase; NPV, negative predictive value; PPV, positive predictive value; RBP, retinol-binding protein; ROC, receiver operating characteristic; UACR, urine albumin-to-creatinine ratio; UAlb, urinary albumin.

**Figure 3:**
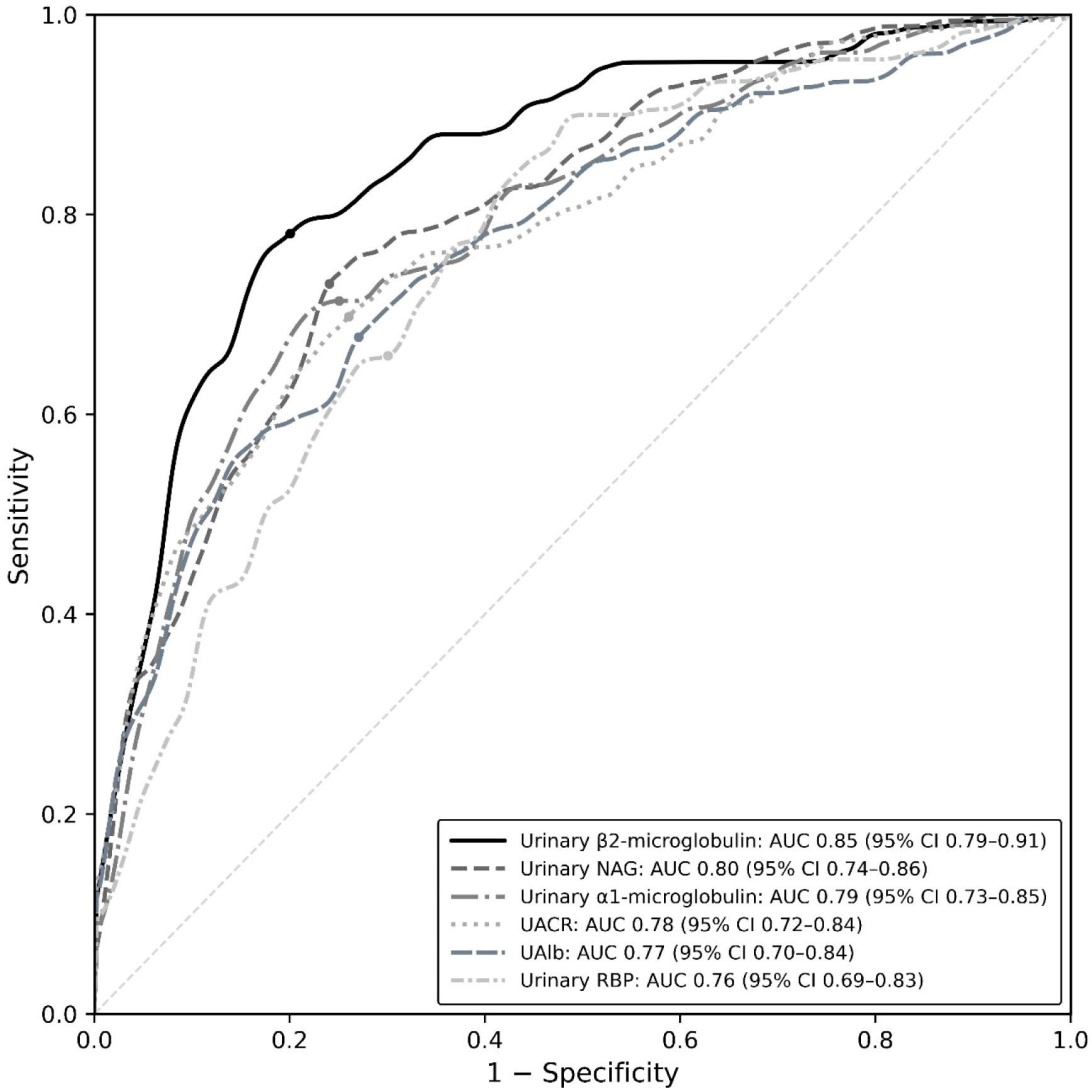
Receiver operating characteristic curves of single urinary biomarkers for distinguishing early-stage (G1–G2) from advanced-stage (G3–G5) kidney injury, showing that urinary β2-microglobulin had the highest discriminative value, followed by urinary N-acetyl-β-D-glucosaminidase, urinary α1-microglobulin, UACR, urinary albumin, and urinary retinol-binding protein.

For optional stage discrimination (early G1–G2 versus advanced G3–G5), tubular protein handling markers exhibited comparatively stronger performance than albumin-based measures. Urinary β2-microglobulin achieved the highest AUC (0.85, 95% CI 0.79–0.91; P < 0.001), with sensitivity of 78.0% and specificity of 80.0% at a cutoff of 1.60 mg/g creatinine. Urinary NAG and urinary α1-microglobulin showed moderate stage discrimination (AUC 0.80 and 0.79; both P < 0.001), while UACR and urinary albumin yielded slightly lower AUCs (0.78 and 0.77). With an advanced-stage prevalence of 25%, NPVs were consistently high (86.1%–91.6%), whereas PPVs were lower (42.3%–56.5%), reflecting the lower prevalence of advanced disease (**Table 6**; **Figure 3**).

**Table 6:** Stage discrimination performance for distinguishing early (G1–G2) versus advanced (G3–G5) kidney injury.

Biomarker	AUC (95% CI)	P for AUC > 0.50	Optimal cutoff (Youden)	Sensitivity, %	Specificity, %	PPV, %	NPV, %
Urinary β2-microglobulin, mg/g creatinine	0.85 (0.79–0.91)	<0.001	1.60 mg/g	78.0	80.0	56.5	91.6
Urinary NAG, U/g creatinine	0.80 (0.74–0.86)	<0.001	3.00 U/g	75.0	76.0	51.0	90.1
Urinary α1-microglobulin, mg/g creatinine	0.79 (0.73–0.85)	<0.001	2.20 mg/g	72.0	75.0	49.0	88.9
UACR, mg/g	0.78 (0.72–0.84)	<0.001	120 mg/g	70.0	74.0	47.3	88.1
Urinary albumin (UAlb), mg/L	0.77 (0.70–0.84)	<0.001	90 mg/L	68.0	73.0	45.6	87.3
Urinary RBP, mg/g creatinine	0.76 (0.69–0.83)	<0.001	1.20 mg/g	66.0	70.0	42.3	86.1

AUC, area under the receiver operating characteristic curve; DN, diabetic nephropathy; NAG, N-acetyl-β-D-glucosaminidase; NPV, negative predictive value; PPV, positive predictive value; RBP, retinol-binding protein; ROC, receiver operating characteristic; UACR, urine albumin-to-creatinine ratio; UAlb, urinary albumin.

### 3.7 Diagnostic performance of combined models and incremental value beyond single biomarkers

Using UACR alone yielded an AUC of 0.93 (95% CI 0.90–0.96), with 89.0% sensitivity and 90.0% specificity at the Youden-derived threshold. A glomerular-only model (UACR plus urinary albumin) provided a numerically higher AUC (0.94, 95% CI 0.91–0.97), but the AUC increment relative to UACR alone was not statistically significant by DeLong testing (Z = 1.30, P = 0.194). A tubular-only model comprising urinary β2-microglobulin, urinary NAG, urinary α1-microglobulin, and urinary RBP achieved an AUC of 0.91 (95% CI 0.88–0.94), which was modestly lower than UACR alone (DeLong Z = −2.08, P = 0.038). In contrast, the combined tubular–glomerular model (UACR + urinary β2-microglobulin + urinary NAG) demonstrated higher discrimination (AUC 0.96, 95% CI 0.94–0.98) and significantly outperformed the UACR-only model (DeLong Z = 2.74, P = 0.006). The extended model that additionally incorporated clinical covariates (diabetes duration, HbA1c, and hypertension) further increased AUC to 0.97 (95% CI 0.95–0.99) and showed significant incremental discrimination over UACR alone (DeLong Z = 3.41, P < 0.001). Calibration metrics suggested adequate agreement between predicted and observed probabilities for the combined tubular–glomerular and extended models, with non-significant Hosmer–Lemeshow tests (P > 0.60) and lower Brier scores than the single-marker model (0.089–0.082 vs 0.112), indicating improved overall prediction accuracy (**Table 7**). Variance inflation factor analysis indicated no meaningful multicollinearity in the primary combined biomarker models, with all VIFs below 2.5 and well under the prespecified threshold of 5 (**Supplementary Table 2**). Bootstrap internal validation showed only modest optimism in model performance. Although the apparent AUCs of the glomerular-only, tubular-only, combined tubular–glomerular, and extended models were slightly reduced after optimism correction, discrimination remained high overall. Specifically, the optimism-corrected AUCs were 0.93 for the glomerular-only model, 0.89 for the tubular-only model, 0.95 for the combined tubular–glomerular model, and 0.95 for the extended model. Calibration also remained acceptable, with optimism-corrected calibration slopes close to 1.0 and only small increases in corrected Brier scores. These findings suggest only modest optimism and limited overfitting, supporting the internal stability of the primary diagnostic models (**Supplementary Table 3**).

**Table 7:** Diagnostic performance of combined models for DN identification and comparison with single-biomarker models.

Model	Components	AUC (95% CI)	Sensitivity, %	Specificity, %	PPV, %	NPV, %	Hosmer–Lemeshow χ² (P)	Brier score	ΔAUC vs UACR (DeLong Z)	P value
Single marker	UACR	0.93 (0.90–0.96)	89.0	90.0	89.9	89.1	7.12 (0.524)	0.112	Reference	—
Combined (glomerular)	UACR + UAlb	0.94 (0.91–0.97)	90.0	90.0	90.0	90.0	6.88 (0.550)	0.104	1.30	0.194
Combined (tubular)	β2-MG + NAG + α1-MG + RBP	0.91 (0.88–0.94)	85.0	87.0	86.7	85.4	8.41 (0.393)	0.127	−2.08	0.038
Combined (tubular + glomerular)	UACR + β2-MG + NAG	0.96 (0.94–0.98)	92.0	91.0	91.1	91.9	5.94 (0.654)	0.089	2.74	0.006
Extended (add clinical)	UACR + β2-MG + NAG + diabetes duration + HbA1c + hypertension	0.97 (0.95–0.99)	93.0	92.0	92.1	92.9	6.17 (0.629)	0.082	3.41	<0.001

AUC, area under the receiver operating characteristic curve; β2-MG, urinary β2-microglobulin; DN, diabetic nephropathy; NAG, N-acetyl-β-D-glucosaminidase; NPV, negative predictive value; PPV, positive predictive value; RBP, retinol-binding protein; UACR, urine albumin-to-creatinine ratio; UAlb, urinary albumin.

### 3.8 Robustness of findings in subgroup and sensitivity analyses

In prespecified subgroup analyses stratified by age, sex, diabetes duration, hypertension status, and renin–angiotensin system blocker use, the combined biomarker model (UACR + urinary β2-microglobulin + urinary NAG) maintained high discrimination for DN across all strata, with AUCs ranging from 0.95 to 0.96 (all P < 0.001). Formal tests for heterogeneity did not indicate statistically significant differences in AUC across age groups (P for interaction = 0.410), sex (P for interaction = 0.620), diabetes duration strata (P for interaction = 0.330), hypertension status (P for interaction = 0.540), or RAS blocker use (P for interaction = 0.270) (**Supplementary Table 4**).

Sensitivity analyses designed to evaluate robustness to potential confounding conditions yielded consistent results. After excluding admissions with infection or sepsis, the combined model retained an AUC of 0.96 (95% CI 0.94–0.98), which did not differ from the primary analysis by DeLong testing (Z = 0.28, P = 0.781). Excluding patients with extreme serum creatinine values similarly preserved discrimination (AUC 0.96, 95% CI 0.94–0.98; Z = 0.19, P = 0.850). When both exclusion criteria were applied simultaneously, the AUC remained high (0.95, 95% CI 0.93–0.98) and was not statistically different from the primary estimate (Z = −0.64, P = 0.522). Collectively, these subgroup and sensitivity analyses suggest that the diagnostic performance of the combined tubular–glomerular biomarker model was robust across major demographic and clinical strata (**Supplementary Table 5**).

To further address potential residual confounding arising from baseline differences in clinical characteristics and background medication exposure that were not included in the matching procedure, we performed fully adjusted sensitivity analyses incorporating age, sex, body mass index, diabetes duration, HbA1c, hypertension, renin–angiotensin system blocker use, sodium–glucose cotransporter-2 inhibitor use, glucagon-like peptide-1 receptor agonist use, statin use, and insulin use. After this additional adjustment, UACR, urinary β2-microglobulin, urinary NAG, and the combined biomarker score each remained independently associated with DN when added to the covariate-only model (all P < 0.001). Compared with the covariate-only model (AUC 0.79, 95% CI 0.74–0.84), discrimination improved substantially after addition of UACR (AUC 0.95, 95% CI 0.93–0.97), urinary β2-microglobulin (AUC 0.90, 95% CI 0.87–0.93), urinary NAG (AUC 0.89, 95% CI 0.85–0.92), and especially the combined biomarker score (AUC 0.97, 95% CI 0.95–0.98; all P for ΔAUC < 0.001), with adequate calibration preserved (**Supplementary Table 6**).

### 3.9 *Post hoc* assessment of sample size adequacy for model development

For the binary outcome of DN status, the effective sample size was 160, defined as the smaller of the number of events and non-events. The glomerular-only model included 2 predictor parameters, the tubular-only model 4 predictor parameters, the combined tubular–glomerular model 3 predictor parameters, and the extended model 6 predictor parameters. Accordingly, the events per predictor parameter were 80.0, 40.0, 53.3, and 26.7, respectively, with identical values for non-events per predictor parameter. The total sample size per predictor parameter was 160.0, 80.0, 106.7, and 53.3, respectively. All models therefore exceeded the conventional benchmark of 10 events per predictor parameter by a substantial margin. Together with the small optimism observed on bootstrap internal validation, these findings suggest that the available sample size was sufficient to support the complexity of the primary diagnostic models and that major overfitting due to model complexity alone was unlikely.

## 4 Discussion

This retrospective matched study evaluated the stage-wise distribution and diagnostic utility of clinically accessible tubular and glomerular biomarkers across eGFR categories (G1–G5) in patients with type 2 diabetes, comparing those with DN with controls without nephropathy. The principal findings were fourfold. First, conventional indices of kidney dysfunction, including serum creatinine and blood urea nitrogen, increased progressively across worsening eGFR stages with significant monotonic trends, supporting the internal consistency of eGFR-based staging. Second, markers reflecting glomerular barrier injury and permeability, including UACR, urinary albumin, urinary transferrin, and urinary IgG, clearly distinguished DN from controls even at preserved eGFR (G1) and generally increased with worsening kidney dysfunction in DN, consistent with progressive glomerular injury. Third, commonly used tubular biomarkers, including urinary N-acetyl-β-D-glucosaminidase, urinary β2-microglobulin, urinary α1-microglobulin, and urinary retinol-binding protein, were already elevated in DN at early stages and showed significant stage-related trends with declining eGFR, supporting an important tubular component across the course of DN. Fourth, diagnostic performance analyses indicated that albuminuria-based measures provided the highest single-marker discrimination for DN, whereas tubular markers contributed comparatively more to differentiating early from advanced eGFR stages; integration of tubular and glomerular markers improved discrimination beyond UACR alone and remained stable across prespecified subgroups.

The present findings indicate that routinely available urinary biomarkers reflecting both glomerular and tubular injury provide clinically meaningful and biologically complementary information for identifying DN in hospitalized patients with type 2 diabetes. Among single markers, glomerular indices showed the strongest overall discrimination, with UACR achieving the highest AUC (0.93), followed closely by urinary albumin (0.91), which underscores the central diagnostic value of albuminuric injury in established diabetic renal disease. At the same time, several tubular biomarkers, particularly urinary β2-microglobulin and urinary NAG, also demonstrated robust diagnostic performance and were already significantly elevated in the G1 subgroup, when eGFR remained preserved. This pattern suggests that tubular injury is not merely a late consequence of declining kidney function but is detectable early in the disease course and may provide information beyond conventional albumin-centered assessment (12, 13). The stage-wise analyses further strengthen this interpretation. In the DN group, both glomerular and tubular biomarkers showed clear monotonic increases with worsening eGFR, whereas such trends were absent in controls, indicating that these markers capture progressive pathophysiologic changes linked to diabetic kidney involvement rather than nonspecific variation across kidney function strata. Of note, the strong early-stage separation between DN and controls for urinary transferrin, urinary IgG, urinary β2-microglobulin, and urinary NAG supports the view that abnormalities in glomerular barrier selectivity and proximal tubular protein handling may emerge concurrently and may jointly characterize early renal injury in diabetes (14, 15). Taken together, these results support a multidimensional interpretation of DN in which albuminuria remains highly informative, but adjunctive tubular biomarkers enhance disease characterization by reflecting additional compartments of injury and may improve recognition of clinically relevant renal involvement before substantial loss of filtration function becomes apparent (16).

A second important finding is that integrating glomerular and tubular biomarkers improved diagnostic discrimination beyond single-marker strategies, with good calibration and consistent performance across multiple robustness analyses. Although the glomerular-only model modestly increased the AUC relative to UACR alone, this increment was not statistically significant, indicating that adding another closely related glomerular marker may offer limited incremental value. In contrast, the combined tubular–glomerular model incorporating UACR, urinary β2-microglobulin, and urinary NAG significantly outperformed the UACR-only model and achieved a high AUC of 0.96, while the extended model including diabetes duration, HbA1c, and hypertension further increased the AUC to 0.97. These findings suggest that combining biomarkers from distinct pathophysiologic domains, together with selected clinical variables, yields a more informative representation of DN than reliance on albuminuria alone. Importantly, this improved discrimination was not accompanied by evidence of model instability. Multicollinearity was low, calibration metrics remained acceptable, and bootstrap validation showed only modest optimism, supporting the internal consistency of the models. Moreover, the combined model retained high performance across predefined subgroups and after exclusion-based sensitivity analyses, and the medication-adjusted sensitivity models demonstrated that the associations of UACR, urinary β2-microglobulin, urinary NAG, and the combined score with DN persisted after accounting for important clinical and treatment-related covariates (17, 18). From a clinical perspective, these results suggest that a pragmatic panel composed of routinely measured urinary markers may offer a feasible and translationally relevant approach for risk stratification in real-world settings (19, 20). Rather than replacing conventional markers, such a strategy may refine case identification and staging by integrating complementary signals of glomerular permeability disturbance and tubular dysfunction within a single diagnostic framework (21, 22).

In our study, most tubular biomarkers rose progressively across eGFR stages, whereas urinary retinol-binding protein showed attenuation at G5, indicating that urinary biomarker trajectories in advanced diabetic kidney disease may be non-linear (23). A plausible explanation is that, in end-stage renal dysfunction, extensive tubular atrophy and interstitial fibrosis, markedly reduced nephron mass, and impaired urine concentrating and excretory capacity may limit the urinary release or measurable excretion of certain low-molecular-weight proteins (24). In this setting, urinary biomarker levels may reflect not only injury severity but also residual tubular handling capacity, which may account for plateauing or partial decline despite ongoing structural damage (25). In addition, biomarker behavior may vary across biological and clinical contexts (26). Sex- and ethnicity-related differences in albuminuria patterns and DKD progression have been reported, raising the possibility that biomarker distributions and optimal thresholds may not be fully generalizable across populations (27). Treatment setting may also contribute to variability, particularly when background renoprotective therapy modifies albuminuria or tubular stress. Notably, in our cohort the combined model remained robust across sex and RAS-blocker strata, suggesting acceptable internal consistency, although external validation in broader ethnic and therapeutic settings remains necessary.

Several limitations should be acknowledged. First, as a retrospective multicenter study restricted to hospitalized patients, the cohort may overrepresent individuals with more severe disease and more complex clinical profiles, which may limit generalizability to outpatient or earlier-stage populations. Second, although matching was performed, residual confounding cannot be fully excluded because of incomplete covariate capture inherent to retrospective data. Third, DN classification was based on operational clinical criteria and diagnostic coding rather than uniform histopathologic confirmation, introducing potential misclassification. Fourth, despite prespecified sampling rules, single-measurement biomarker assessment remains susceptible to short-term biological variability. Fifth, the small G5 subgroup may have reduced the precision of stage-specific estimates in advanced kidney dysfunction. Sixth, creatinine-indexed urinary biomarkers may still be influenced by factors such as age, sex, muscle mass, and kidney function. Finally, the cutoff values derived from the present cohort are sample-dependent, and the subgroup analyses should be interpreted as exploratory. Accordingly, external validation in broader and more diverse populations is warranted. Future studies should prioritize prospective, longitudinal, and externally validated designs with repeated standardized urine collections, broader recruitment across clinical settings, and inclusion of more diverse ethnic and therapeutic populations. Further work should also evaluate longitudinal renal and survival outcomes and determine whether integrating emerging urinary biomarkers or multi-omics approaches with clinically accessible tubular and glomerular markers can further improve diagnostic discrimination, biological stratification, and risk prediction in diabetic kidney disease.

## 5 Conclusion

In this matched cohort of hospitalized patients with type 2 diabetes, both glomerular and tubular urinary biomarkers differentiated DN from controls without nephropathy across eGFR stages, with significant separation evident even in G1. UACR showed the highest single-biomarker discrimination for DN, whereas tubular biomarkers better distinguished early from advanced kidney dysfunction. A combined tubular–glomerular model integrating UACR, urinary β2-microglobulin, and urinary NAG improved discrimination beyond UACR alone and remained robust in prespecified subgroup and sensitivity analyses.

## Data Availability

The raw data supporting the conclusions of this article will be made available by the authors, without undue reservation.
