# Supplementary material for: Diagnostic value of tubular and glomerular biomarkers across different stages of kidney injury in patients with type 2 diabetic nephropathy
Source: Front Endocrinol (Lausanne). 2026 Jun 18;17:1802069. doi: 10.3389/fendo.2026.1802069 (PMC13322808; doi:10.3389/fendo.2026.1802069)
Supplement: Supplementary file 1 [file Table1.docx]

Supplementary Table S1. Assay methods, analytical precision, and inter-laboratory consistency of the primary urinary biomarkers

| Biomarker | Assay method | Reagent / platform | Within-assay CV, % | Between-assay CV, % | Consistency notes |
| --- | --- | --- | --- | --- | --- |
| UACR | Urine albumin by immunoturbidimetry; urine creatinine by enzymatic colorimetric assay; ratio calculated | Same manufacturer-matched urine albumin and creatinine reagents on comparable automated chemistry analyzers | 3.5 | 5.8 | Harmonized calibration and reporting units across centers; ratio derived from standardized albumin and creatinine measurements |
| Urinary albumin (UAlb) | Immunoturbidimetric assay | Same manufacturer-matched urine protein reagent set on comparable automated chemistry analyzers | 3.2 | 5.1 | Same or equivalent calibrators and QC levels used across centers |
| Urinary transferrin (uTRF) | Immunoturbidimetric assay | Same manufacturer-matched urine protein reagent set on comparable automated chemistry analyzers | 4.6 | 8.2 | Harmonized analytical workflow; no center-specific recalibration issues identified |
| Urinary immunoglobulin G (uIgG) | Immunoturbidimetric assay | Same manufacturer-matched urine protein reagent set on comparable automated chemistry analyzers | 4.9 | 8.8 | Measured under the same urine protein platform framework |
| Urinary N-acetyl-β-D-glucosaminidase (NAG) | Enzymatic colorimetric / spectrophotometric assay | Same manufacturer-matched enzymatic reagent kit on comparable automated chemistry analyzers | 3.7 | 6.8 | Standardized substrate-based enzymatic method with routine two-level QC |
| Urinary β2-microglobulin | Immunoturbidimetric assay | Same manufacturer-matched low-molecular-weight protein reagent set on comparable automated platforms | 4.4 | 8 | Sample handling standardized across centers; alkaline handling/storage precautions applied when required |
| Urinary α1-microglobulin | Immunonephelometric or immunoturbidimetric assay | Same manufacturer-matched low-molecular-weight protein reagent set on comparable automated platforms | 5.9 | 11.3 | Same assay family and QC procedures across centers |
| Urinary retinol-binding protein (RBP) | Immunoturbidimetric assay | Same manufacturer-matched low-molecular-weight protein reagent set on comparable automated platforms | 4.9 | 7 | Harmonized urine protein workflow; no material inter-laboratory inconsistency detected in QC review |

CV, coefficient of variation; NAG, N-acetyl-β-D-glucosaminidase; RBP, retinol-binding protein; UACR, urine albumin-to-creatinine ratio.

Supplementary Table S2. Variance inflation factors for biomarker predictors included in the primary combined biomarker models

| Predictor | Glomerular-only model (UACR + UAlb) | Tubular-only model (β2-MG + NAG + α1-MG + RBP) | Combined tubular–glomerular model (UACR + β2-MG + NAG) | Extended model |
| --- | --- | --- | --- | --- |
| UACR | 2.18 | — | 1.43 | 1.47 |
| UAlb | 2.18 | — | — | — |
| Urinary β2-microglobulin | — | 2.34 | 1.36 | 1.39 |
| Urinary NAG | — | 1.88 | 1.29 | 1.31 |
| Urinary α1-microglobulin | — | 2.11 | — | — |
| Urinary RBP | — | 1.74 | — | — |

NAG, N-acetyl-β-D-glucosaminidase; RBP, retinol-binding protein; UACR, urine albumin-to-creatinine ratio; UAlb, urinary albumin; VIF, variance inflation factor.

Supplementary Table S3. Bootstrap internal validation of the primary combined biomarker models

| Model | Apparent AUC | Optimism in AUC | Optimism-corrected AUC | Apparent Brier score | Optimism-corrected Brier score | Optimism-corrected calibration slope |
| --- | --- | --- | --- | --- | --- | --- |
| Glomerular-only model (UACR + UAlb) | 0.94 | 0.01 | 0.93 | 0.104 | 0.109 | 0.96 |
| Tubular-only model (β2-MG + NAG + α1-MG + RBP) | 0.91 | 0.02 | 0.89 | 0.127 | 0.134 | 0.92 |
| Combined tubular–glomerular model (UACR + β2-MG + NAG) | 0.96 | 0.01 | 0.95 | 0.089 | 0.095 | 0.95 |
| Extended model (UACR + β2-MG + NAG + diabetes duration + HbA1c + hypertension) | 0.97 | 0.02 | 0.95 | 0.082 | 0.09 | 0.91 |

AUC, area under the receiver operating characteristic curve; NAG, urinary N-acetyl-β-D-glucosaminidase; UACR, urine albumin-to-creatinine ratio; UAlb, urinary albumin; β2-MG, urinary β2-microglobulin; RBP, retinol-binding protein.

Note: Internal validation was performed using 1,000 bootstrap resamples.

Supplementary Table S4. Prespecified Subgroup Analyses for DN Identification Using the Combined Model (UACR + Urinary β2-Microglobulin + Urinary NAG)

| Subgroup | n (DN / Control) | AUC (95% CI) | P value (AUC > 0.50) | P for interaction |
| --- | --- | --- | --- | --- |
| Age, years |  |  |  |  |
| <65 | 160 (80 / 80) | 0.95 (0.91–0.98) | <0.001 | 0.410 |
| ≥65 | 160 (80 / 80) | 0.96 (0.92–0.99) | <0.001 |  |
| Sex |  |  |  |  |
| Male | 194 (98 / 96) | 0.96 (0.93–0.98) | <0.001 | 0.620 |
| Female | 126 (62 / 64) | 0.95 (0.91–0.99) | <0.001 |  |
| Diabetes duration, years |  |  |  |  |
| <10 | 162 (80 / 82) | 0.95 (0.91–0.98) | <0.001 | 0.330 |
| ≥10 | 158 (80 / 78) | 0.96 (0.93–0.99) | <0.001 |  |
| Hypertension |  |  |  |  |
| No | 106 (44 / 62) | 0.95 (0.90–0.99) | <0.001 | 0.540 |
| Yes | 214 (116 / 98) | 0.96 (0.93–0.98) | <0.001 |  |
| RAS blocker use |  |  |  |  |
| No | 154 (56 / 98) | 0.95 (0.92–0.98) | <0.001 | 0.270 |
| Yes | 166 (104 / 62) | 0.96 (0.93–0.99) | <0.001 |  |

AUC, area under the receiver operating characteristic curve; DN, diabetic nephropathy; NAG, N-acetyl-β-D-glucosaminidase; RAS, renin–angiotensin system; UACR, urine albumin-to-creatinine ratio.

Supplementary Table S5. Sensitivity Analyses Assessing Robustness of the Combined Model for DN Identification.

| Analysis | Exclusion criteria | Remaining n (DN / Control) | AUC (95% CI) | DeLong test vs primary (Z) | P value |
| --- | --- | --- | --- | --- | --- |
| Primary analysis | None | 320 (160 / 160) | 0.96 (0.94–0.98) | Reference | — |
| Sensitivity 1 | Excluded admissions with infection or sepsis | 304 (152 / 152) | 0.96 (0.94–0.98) | 0.28 | 0.781 |
| Sensitivity 2 | Excluded extreme Scr values (top 1% within cohort) | 316 (158 / 158) | 0.96 (0.94–0.98) | 0.19 | 0.850 |
| Sensitivity 3 | Excluded both infection/sepsis and extreme Scr | 300 (150 / 150) | 0.95 (0.93–0.98) | −0.64 | 0.522 |

AUC, area under the receiver operating characteristic curve; DN, diabetic nephropathy; NAG, N-acetyl-β-D-glucosaminidase; Scr, serum creatinine; UACR, urine albumin-to-creatinine ratio.

Supplementary Table S6. Fully adjusted sensitivity analyses for identifying DN

| Model | aOR per 1-SD increase (95% CI) | P value | AUC (95% CI) | Sensitivity, % | Specificity, % | Hosmer–Lemeshow P | Brier score | ΔAUC vs covariate-only | P for ΔAUC |
| --- | --- | --- | --- | --- | --- | --- | --- | --- | --- |
| Covariate-only model* | — | — | 0.79 (0.74–0.84) | 72 | 74 | 0.483 | 0.166 | Reference | — |
| UACR + covariates | 4.28 (2.98–6.15) | <0.001 | 0.95 (0.93–0.97) | 90 | 91 | 0.624 | 0.095 | 0.16 | <0.001 |
| Urinary β2-microglobulin + covariates | 2.81 (1.99–3.98) | <0.001 | 0.90 (0.87–0.93) | 83 | 84 | 0.593 | 0.123 | 0.11 | <0.001 |
| Urinary NAG + covariates | 2.46 (1.79–3.39) | <0.001 | 0.89 (0.85–0.92) | 81 | 83 | 0.611 | 0.131 | 0.1 | <0.001 |
| Combined biomarker score† + covariates | 6.55 (4.34–9.88) | <0.001 | 0.97 (0.95–0.98) | 93 | 92 | 0.652 | 0.079 | 0.18 | <0.001 |

aOR, adjusted odds ratio; AUC, area under the receiver operating characteristic curve; DN, diabetic nephropathy; NAG, N-acetyl-β-D-glucosaminidase; UACR, urine albumin-to-creatinine ratio.

* Covariates included age, sex, body mass index, diabetes duration, HbA1c, hypertension, renin–angiotensin system blocker use, sodium–glucose cotransporter-2 inhibitor use, glucagon-like peptide-1 receptor agonist use, statin use, and insulin use.

† Combined biomarker score was derived from the primary tubular–glomerular model including UACR, urinary β2-microglobulin, and urinary NAG. Biomarkers were log-transformed as appropriate and standardized before modeling; aORs are expressed per 1-standard-deviation increase.
